# Integrating nurse practitioners into primary care: policy considerations from a Canadian province

**DOI:** 10.1186/s12875-020-01318-3

**Published:** 2020-12-04

**Authors:** Stacey Black, Raad Fadaak, Myles Leslie

**Affiliations:** The School of Public Policy, University of Calgary, Calgary, Alberta Canada

**Keywords:** Nurse practitioners, Primary care, Policy, Funding, Role, Integration, Collaboration

## Abstract

**Background:**

The integration of nurse practitioners (NPs) into primary care health teams has been an object of interest for policy makers seeking to achieve the goals of improving care, increasing access, and lowering cost. The province of Alberta in Canada recently introduced a policy aimed at integrating NPs into existing primary care delivery structures. This qualitative research sought to understand how that policy – the NP Support Program (NPSP) – was viewed by key stakeholders and to draw out policy lessons.

**Methods:**

Fifteen semi-structured interviews with NPs and other stakeholders in Alberta’s primary care system were conducted, recorded, transcribed and analyzed using the interpretive description method.

**Results:**

Stakeholders predominantly felt the NPSP would not change the status quo of limited practice opportunities and the resulting underutilization of primary care NPs in the province. Participants attributed low levels of NP integration into the primary care system to: 1) financial viability issues that directly impacted NPs, physicians, and primary care networks (PCNs); 2) policy issues related to the NPSP’s reliance on PCNs as employers, and a requirement that NPs panel patients; and 3) governance issues in which NPs are not afforded sufficient authority over their role or how the key concept of ‘care team’ is defined and operationalized.

**Conclusions:**

In general, stakeholders did not see the NPSP as a long-term solution for increasing NP integration into the province’s primary care system. Policy adjustments that enable NPs to access funding not only from within but also outside PCNs, and modifications to allow greater NP input into how their role is utilized would likely improve the NPSP’s ability to reach its goals.

## Background

The integration of nurse practitioners (NPs) into primary care has been viewed as a solution to shortages of doctors [1], and a tool for improving patient access to care and lowering costs [2]. It has also been suggested that the integration of NPs into community-based care delivery is critical to accomplishing the transformation of primary care into primary health care (PHC) [3, 4] - a transformation that has itself been linked to improved care, improved outcomes, and lowered costs [5–8]. The shift to PHC is one towards prevention, health, wellness and the successful management of chronic disease [9–14], accomplished through team-based care [15]. The integration of NPs into PHC-focused teams, and their integration into healthcare systems more broadly has been advocated for in a range of policy environments, including both Canada and the United States [16–19]. Despite this alignment with transformation principles and much policy enthusiasm, the utilization of NPs in Canadian primary care has been inconsistent [17].

To better understand the factors behind the inconsistent utilization of NPs, this paper presents one Canadian province’s recent and ongoing efforts to increase the integration of NPs into its primary care system. We describe the *Nurse Practitioner Support Program* (NPSP^1^) in Alberta, Canada, and present qualitative data from interviews with stakeholders. These interviews highlight the challenges to achieving the goals outlined in the NPSP and to NP integration in the province’s primary care environment. The data we present here provide broader comparative learnings for other jurisdictions contemplating policies to support greater NP integration into their primary care systems.

NPs in Alberta are registered nurses (RNs) that have: worked at least 4500 RN hours; completed a recognized NP educational program; and passed a standardized exam specific to their practice area [20]. Drawing on competencies acquired in their Masters-level, clinically focused education [21] NPs enjoy a wider scope of practice than RNs, including the ability to: conduct advanced health assessments; order and interpret diagnostic tests; diagnose, treat, and perform advanced interventions; prescribe medications; monitor patient outcomes; and refer patients to other professionals as required [22]. Currently all provinces in Canada have legislation regulating the NP scope of practice [2, 23]. In the province of Alberta, the Health Professions Act [24] and the Registered Nurses Profession Regulation [25] requires NPs be registered with the College and Association of Registered Nurses of Alberta (CARNA). CARNA requirements govern maintenance of an NP’s license to practice [22]. Despite a regulatory presence in colleges and legislation across Canada, [2] funding and payment reform to support NP integration have proved to be barriers to practical implementation of the role [26].

The NPSP was not the Alberta government’s first attempt at increasing NP participation in primary care [27]. In 2012, the province announced an increased role for NPs with the introduction of family care clinics [28]. Seen as costly and opposed by some members of the medical community [29, 30], the family care clinic concept did not successfully navigate a change in political leadership [31, 32]. Only 3 out of the 140 clinics envisioned were ultimately built [31].

In April 2019, with a commitment to team-based care^2^ and its advantages [33] well-articulated in the provincial primary care strategy [4], the province sought to increase the relatively low count of NPs in primary care by introducing the NPSP [17]. In Alberta the majority of registered NPs – 72% in 2018 – were employed in acute care or specialty out-patient clinics [17]. In other Provinces the majority of NPs work in primary care and community settings with only 24% working in hospitals [35]. The NPSP thus aimed to increase the use of NPs in Alberta’s primary care system, part of a broader provincial government priority to improve access, safety, quality, and continuity of primary care [17].

The NPSP allows the province’s Primary Care Networks (PCNs) to apply for NP-specific funding, with the aim of increasing NP integration into primary care. PCNs are intermediary organizations positioned between the government and the front lines of primary care, and as envisioned by the NPSP, are the primary agents for primary care NP integration in the province. Originally created in 2003, PCNs are funded by the Government of Alberta’s Ministry of Health and formed through a joint venture arrangement between Alberta Health Services (AHS; the single health authority and service provider for the province) and primary care physicians who opt-in, signing a contract to become members of a PCN [36].

In Alberta, individual physicians are generally independent small-business owners, with the province functioning almost exclusively on a Fee-For-Service (FFS) model [37–40]. Physicians can however arrange an alternative compensation method with the government if they choose [41]. These compensation options are available to primary care physicians whether they choose to join a PCN or not. As noted above, primary care physicians can opt to join a PCN, which generates money for their respective PCN. This per capita funding for the PCN is in addition to the physician’s regular compensation. For each patient on a PCN-physician’s panel, a PCN receives $62 per year, with this collective per capita money being used to support locally adapted service delivery and team enhancement, as well as pay the PCN’s administrative costs. In addition to implementing team-based care, the PCNs are also responsible for implementing other elements of the Patient Medical Home (PMH) including improved access, patient panelling, use of electronic medical records, and quality improvement [4, 42].

The NPSP positions Alberta’s PCNs as the primary support and implementation mechanism for achieving NP integration into primary care, as part of a broader western Canadian objective of delivering team-based primary care [15]. In light of the province’s relatively low rate of NP uptake in primary care, the NPSP aims to incentivize PCNs to hire more NPs in Alberta and to increase NP integration into primary care teams and achieve primary healthcare transformation [17].

In order to place NPs within the PCN structure, the NPSP introduced changes to the PCN funding formula allowing NPs, and not just physicians, to create a ‘panel’ of patients [43]. Where a panel refers to a set of patients attached to a particular *provider,* a ‘roster’ refers to a set of patients attached to a *group of providers* [43]. The funding generated from an NP’s PCN-based panel of patients (the same $62 per capita) would, unlike with physicians, be earmarked to cover that NP’s salary. In other words, according to the NPSP, if a patient is paneled to an NP, the $62 per capita payment is routed to that NP’s salary which offsets (and decreases) the government’s supplemental top-up, paid to the PCN to support the NP’s annual salary. This supplemental top up is to a maximum of $125,000 [17]. In contrast, if a patient is paneled to a physician, the per capita payments do not reduce the amount the government remunerates that physician based on FFS billings. Instead, a physician bills the government the regular amount *and* the physician’s per capita money is pooled with other PCN physician members’ per capita panel funds and made available to the PCN [17]. This financial arrangement, alongside other factors discussed below, directly impacts the viability of the NPSP policy aimed at integrating NPs into Alberta’s primary care system.

Changes in government often introduce differing priorities with existing publicly funded policies either changed or abandoned. Shortly after the introduction of the NPSP, there was a change in Alberta’s government. However, the integration of NPs into primary care continues to be a priority, with the new government promising NP billing reform [44] and continued support for the NPSP [45]. The urgency of the issue has been emphasized as NPs and their scope of practice have once again come to the forefront as part of the response to the COVID-19 pandemic and the strains it introduced to the primary care system [46].

This paper draws on qualitative interview data to understand NP integration challenges and how a policy like the NPSP is viewed by key stakeholders (NPs, physicians, individuals from nursing and medical professional associations, a patient advocacy group representative, a patient, PCN administrators, and government officials). In the context of the NPSP’s introduction and ongoing interest in NP integration, we interviewed a range of stakeholders seeking to understand the factors that might be limiting the NPSP’s success and ultimately NP integration into Alberta’s primary care environment. Our guiding research questions sought to:

- Identify policy and operational factors that are shaping how Nurse Practitioners (NPs) integrate into Alberta’s PCNs.
- Deepen understanding of the effects that current funding policies and incentivization strategies are having on NP participation in primary care.
- Understand how the policy innovations targeting the integration of NPs into PCNs are perceived by key stakeholders.
- Make evidence-based recommendations regarding policy and operational factors that will help improve NP integration into PCNs and PCN effectiveness.

## Methods

Semi-structured interviews (*n* = 15) were conducted, recorded, transcribed and analyzed using the interpretive description method. Interpretive description is a qualitative, analytical, inductive method of inquiry that focuses on generating practically-applicable knowledge for healthcare issues [47, 48]. We used semi-structured interviews to collect a wide range of perspectives, reflections, and practical knowledge related to the NPSP in Alberta. These interviews were conducted with a range of healthcare providers, decision makers/ influencers, and patient advocates.

Further information is provided in Table 1 below.

**Table 1:** Participants

**Participant Occupation/ Affiliation**	Nursing Leader III	Policy Maker VI	PCN-Member Family Physicians II&XV	Primary Care NPs I, VII, XI, XIV	PCN Administrators XII& IX	Patients X & VIII Patient Advocacy Group V	NP Leader IV	Medical Association Leader XIII
	A senior representative at the province’s professional and regulatory body, (CARNA)^a^	A civil servant from a division of the ministry of health	Two Family physicians that work in primary care	Four NPs that work in primary care	Two PCN Administrators	Patient advocates from the province and a patient who had received care from an NP in the community	A senior executive at the province’s NP Assoc. (NPAA)^b^	A senior representative of the province’s professional and regulatory body, (AMA)^c^
**Number Interviewed**	1	1	2	4	2	3	1	1

^a^College and Association of Registered Nurses of Alberta (CARNA)

^b^Nurse Practitioner Association of Alberta (NPAA)

^c^Alberta Medical Association (AMA)

Recruitment began purposively – with the express aim of including a diversity of perspectives driving our choices [49] – and then shifted to include snowball sampling of individuals through participant referrals [49, 50]. We sent out a recruitment email to potential participants and included research objectives and a template of interview questions. The main focus of the questions involved asking participants for their perception of NP integration in primary care, and their views on the NPSP. Participants are identified in the following pages with the Roman numerals I to XV. In total, we interviewed 15 participants, with this sample composed of different professional backgrounds to ensure potential differences in opinion were considered (See Table 1). Over the course of collecting data from this sample, we found that we had achieved “a realistic range of predictable variance” [51] in the opinions and perspectives that participants were advancing.

The semi-structured interviews were conducted by SB using a guide that was developed iteratively with ML. The guide aimed to draw out rich, nuanced, self-reflective responses from the participants. The interviews ranged in length between 30 to 90 min and were both recorded and transcribed. The data was then analyzed with the assistance of NVivo 12 software using an inductive coding approach aimed at rendering an interpretive description of participants views on NP integration into primary care generally, and via the PCNs and the NPSP specifically.

Our interpretive description approach allowed us to gain insight not just on areas of commonality, but areas of disagreement amongst participants, and with an eye on providing pragmatic suggestions to improve policy in the area [51, 52]. SB and ML analysed the data iteratively, expanding, collapsing and merging themes to arrive at the final analysis. We carried out participant checks on the interpretations presented.

This research obtained ethical approval from the Conjoint Faculties Research Ethics Board at the University of Calgary (REB18–1709). Participants provided written consent.

## Results

Participants described NPs as an underutilized resource in Alberta’s primary care environment. The data from 15 participant interviews attributed this low level of NP integration to a lack of independent practice opportunity and minimal job prospects which, in turn, related to: 1) financial viability issues that impact both NPs, physicians, and PCNs; 2) ineffective policy, and 3) issues with governance.

### A lack of available jobs

As a policy maker participant noted, Alberta is “… under-using [NPs] … especially in primary care” (Participant VI). A PCN executive director described how there had been little progress in integrating NPs into primary care in the preceding decade:

*There were very, very few positions outside of the acute care setting. And I think, in some ways still – like 10 years later, there hasn’t been much progress. (Participant IX).*

The lack of available positions was, in the same participant’s mind, a disservice to both those undertaking NP training and the taxpayers who subsidize that training:

*All of those professional [programs] are generally topped up by government funding in the education stream... I don’t think we were doing NPs a service when they could obtain this education, graduate, and then not have any … positions in the primary care environment [available]. (Participant IX).*

Another participant further elaborated:

*[I]t’s so sad that this cream of the crop bunch [is] being lost [due] to lack of opportunity... what a brain drain! And they’re leaving the province, they’re leaving the country … they are leaving the profession. [W] hat a waste of human resources. (Participant I).*

The lack of job openings and government-funded brain drain to other jurisdictions was viewed by participants as a result of specific financial disincentives that shaped the viability of becoming an NP in the province’s primary care system. These financial viability issues impacted all three of the key stakeholders involved in the NPSP: NPs, physicians, and the PCNs.

### Financial viability: NPs

A range of participants described how the province’s physicians have an assortment of options to fund their practice (FFS, blended capitations, salary). However, the majority of family physicians in Alberta operate as a private business and fund their practice by billing the government FFS. In contrast, NPs – both under the NPSP and prior to its introduction – can *only* be funded as employees (Participant I, XIV, XII), relying on physicians or PCNs having available jobs for them. As one participant noted, independent NP practice “isn’t supported” by current policy arrangements (Participant III).

A policy maker with 20 years experience suggested this was because:

*[T] he compensation systems are not in place to support independent [NP] practice right now (Participant VI).*

This lack of funding options was emphasized by another participant – an NP – who explained how, despite being trained and given authority under legislation to provide certain healthcare services, NPs cannot be paid directly:

*I can’t get paid by the government to offer those services. [T] he regulatory body and the Health Professions Act of Alberta allows me to do it, but I cannot get paid [to provide those services] (Participant XI).*

Unable to be reimbursed as independent practitioners, NPs for the most part currently can either choose to bill patients directly for services or rely on being hired by PCNs or individual physicians as employees. For those who choose to bill outside the publicly funded system, participants noted that financial viability hinges on exploiting a niche for which patients – otherwise accustomed to ‘free’ primary care – are willing to pay out-of-pocket. Without niche practice able to support private billing, financial viability hinges on becoming an employee of an individual physician or a PCN. As we will see later, the governance and power issues inherent in seeking employment from physicians and PCNs are seen as both personally challenging, and detrimental to NPs practicing at their full scope. Beyond these issues, the rate of pay as an employee and lack of alternative options to practice was summed up by one participant as follows:

*A lot of registered nurses (RNs) … ask me, is it worth it? I would say, no. [T]here’s no opportunity [to get a business loan, to bill the government, or get a job.] … [I] f there is opportunity [to get a job], often you make less than a senior RN. (Participant XI).*

A recent report reviewing the province’s health system agreed with this participant’s point, emphasizing the financial disincentive RNs have to take the further education, training, and testing involved to become an NP [53].

If the root cause of low levels of NP integration is tied, from the perspective of NPs, to a lack of billing options and poor financial incentives, financial viability was also an issue for physicians.

### Financial viability: physicians

Under the province’s predominant physician’s FFS billing arrangements, physicians are not encouraged to deploy NP employees to deliver care for which physicians would otherwise be able to bill the government. For a policy-maker participant, the central problem was a missing mechanism for physicians to recover revenue ‘lost’ when an NP provided a service:

*[I]t’s really unclear to family physicians how they would use an NP in their practice. There’s not a mechanism for the physician [and the practice] to be compensated for the services that that NP delivers right now (Participant VI).*

A physician participant described the disincentive of losing revenue to an NP employee in starker terms:

*[I]t’s lucrative [for physicians] to see easy patients because [physicians] get paid fee-for-service. I think a lot of people in primary care don’t want NPs to do that stuff, because [physicians] think it [is] going to impact their bottom line (Participant II).*

Beyond presenting a challenge to the low-effort, high-reward cases at the heart of current physician profitability, participants noted that the NPSP did not provide ways to generate revenue to cover overhead costs. As an NP who ultimately lost their job noted:

*I was basically taking up space where if a physician was in that space, they could bill the government and pay overhead. And the overhead that physicians pay is astronomical (Participant VII).*

Most family physicians in Alberta operate as a private business with revenue generated from billing the government for services provided. NPs cannot bill the government for services they provide and do not receive compensation directly from government. If a physician or a PCN hires an NP, that NP’s salary is paid from the physician’s or PCN’s revenue. As such, NPs do not generate revenue from government to pay overhead. If an NP opened a clinic outside the public system and billed patients directly, the NP would have to pay the same amount of overhead. However, with patients accustomed to publicly funded primary care services, and many NPs wanting to operate within the public system, the inability to cover overhead costs like their physician colleagues proved problematic for most participants.

One physician participant noted a workaround commonly referred to as the ‘whites of the eyes’ billing. This approach involves the physician entering a consulting room where the NP is finishing an appointment just long enough for cursory interaction with the patient. By doing so, this allows the physician employer to bill for the service delivered by the NP employee. As described by the physician:

*I have to kind of just pop my head in and say, “Hi! Any questions? Let me know...” And that’s a bit ridiculous, but I have to pop my head in so I can bill for those patients (Participant XV).*

This ‘whites of the eyes’ billing approach increased revenue to cover the NP’s overhead costs and salary. However, it appeared to be an exception to a general rule where most physicians instead viewed NPs as a financial burden to their business.

### Financial viability: PCNs

Despite being the focal point of the NPSP, the PCNs – as member-driven organizations composed of family physicians – are also disincentivized by the financial realities of integrating NPs. Where the NPSP aims to use per capita revenue generated by the NP’s patient panel to pay the NP salary, the PCNs’ members tend not to see the value proposition. In an extension of their individual concern for an employee delivering services they would otherwise be able to bill for, PCN members often see their organization’s per capita funds better spent elsewhere. A former PCN administrator noted:

*[T] he prevailing belief among docs is:*

*‘We shouldn’t be using PCN money to fund NPs … because we could just [put a physician in that position and] bill FFS [to cover the physician’s salary and overhead] and do what [the NP is] doing and use the [PCN] money for something else like a chronic disease nurse, a social worker, a pharmacist, [etc.]’ (Participant XII).*

Beyond being unpopular amongst member-physicians, NP employees are a challenge for the PCNs themselves given that they come out of the intermediary organization’s bottom line. As another participant noted, from the PCN’s perspective:

*[T] he services that an NP can offer could be offered similarly by a physician and that physician’s compensation would come out of, not the PCN budget, but [the Ministry of Health’s budget] (Participant XI).*

Alongside these financial viability issues for NPs, physicians, and PCNs, participants also identified policy and governance issues that made attractive jobs as a primary care NP rare.

### Ineffective policy

Participants took issue with specific aspects of the NPSP, focusing on the policy’s paneling requirement and its use of the PCNs as its only mechanism for funding and implementation.

#### Requirements for paneling and full scope primary care

While some participants were pleased that the NPSP allowed NPs to panel patients, others were less interested. As 1 NP participant noted:

*I don’t feel the need to have my own patient panel. There are other nurse practitioners who want to have their own patients. So that’s just a personal preference (Participant VII).*

This sense that the policy’s paneling requirement was unnecessarily restrictive was shared by physicians (Participant XV, Participant II). Another NP participant illustrated what they saw as the ineffectiveness of the paneling requirement by making a hypothetical pitch for money to start up a community-based specialty NP practice:

*I’m a nurse practitioner. I have a sub specialization in chronic pain management. There is a high burden of chronic pain in Calgary and it’s under met. The chronic pain center has a 2 year waiting list. I want to start a clinic that deals with chronic pain patients. I want to submit a business plan … [and], I want to submit an expression of interest to have funding to be able to do this. But I can’t do that. It doesn’t exist. Even under the [NPSP] I can’t do it because the premise is full scope, primary care. (Participant XI).*

In the hypothetical pitch, even if an NP were able to identify a specific area that is underserved by physicians, the NPSP’s demand for only full spectrum – which is to say fully empanelled – care would stop the project moving forward because there is no current funding mechanism that would accommodate the participant’s example. In this way, the policy’s panelling requirement and resulting mandate that NPs integrate by providing full scope primary care fails to allow flexibility for NP integration based both on the needs of the community and practitioner preferences.

#### Funding and implementation through the PCNs

Under the terms of the NPSP, a PCN - and only a PCN - has the option to submit an application for this dedicated NP funding. Some participants questioned the prerogative this gives to PCNs over whether to consider integrating NPs at all. As one former PCN administrator noted:

*If physicians are managing it, it’s a little bit like the mice guarding the cheese … Would they really want to give money to NPs and fund their competition? (Participant XII).*

This sense that PCNs should not be the only avenue for NP funding and integration and options beyond PCNs are necessary were widely shared by other participants (Participants I, III, XI, XIV).

How a given PCN and its PCN physician-members view NPs thus has an oversized effect on how the NPSP gets implemented, as PCNs are the only avenue for NPs to qualify for NPSP funding support. As a policy maker noted:

*[W]e’re getting some early indication that [NPs are] also perceived as a threat, and therefore the physicians don’t want them... (Participant VI).*

Even a PCN administrator participant that was very supportive of NP integration into PCNs emphasized that the PCNs ought not to be the *only* implementation mechanism:

*I think we’re a great avenue to support [NP integration]. But I don’t necessarily think we are the only avenue that could (Participant IX).*

Using PCNs as the exclusive avenue for primary care NP integration was considered a major limitation by many participants. Beyond the need for options, there were deep concerns about the governance and authority structures that are embedded in the PCNs, and their impact on NP integration into Alberta’s primary care environment.

### Inappropriate governance

Both NP and non-NP participants believed that positioning the PCNs as the sole implementation mechanism for the NPSP was highly problematic. Their concerns centered on the fact that PCNs were “controlled by physicians” (Participant III), “physician-centric” (Participant I) (Participant IX), “led by family physicians” (Participant XIV), and “physician-led” (Participant VI).

As 1 NP described it, choosing the PCNs to advance NP integration was one in which the government was essentially “asking another profession – which is physicians - to develop the role of NPs.” This was not only seen as “inappropriate”, but meant the NPSP was “flawed by design” because it, “leaves the decision to physicians to integrate [NPs]; how to integrate them; where to integrate them” (Participant XI).

Further elaborating, the NP emphasized the power imbalance that comes with being an employee rather than a member of a PCN:

*[NPs] can only be an employee of the PCN [not a full member like physicians] … so you’re missing the nursing voice at the table … How you’re deployed, where are you deployed, how you’re utilized. I don’t have any control (Participant XI).*

This loss of input and control was felt keenly by most NP participants. For them, the NPSP supports an inappropriate form of governance and dysfunctional form of team-based care. For one participant, there was a key difference between a team that was working together collegially, and a team that was built around doing work for physicians. This participant described the difference as being one between:

*People who are willing to work actually in a team – [and people who] want a team to work for them. Completely different (Participant I).*

When team was defined collegially, and so governance hierarchies were flattened, not just NP integration, but reported job and patient satisfaction improved. Under these conditions the employee-NP model was viable from a governance standpoint as much as it still suffered from financial challenges. Illustrating this, an NP participant described a period of collegial physician-NP teamwork at a PCN where they worked:

*[W] e called it the Dream Team … the patients were really happy … It [was] the best job of my whole life … And we co-referred, we shared, we had hallway consults – it was incredibly dynamic. The patients got what they want [ed] … our job satisfaction was like a hundred percent … And then, the medical director [of the PCN] came back, and insisted on a hierarchy. And we all got sort of dispersed, and we weren’t allowed to eat lunch together … And almost everybody either quit or was let go (Participant I).*

Where anxieties about overlapping scopes of practice and expertise, as well as financial viability, had briefly been set aside, they returned with the medical director who had the authority to re-impose old hierarchies. With these hierarchies came a revised definition of team. As a concept it shifted away from a collegial levelling and towards treating employee NPs as tools for greater physician productivity. As the social distinctions between the professions were reasserted, and the governance of NPs by physicians became the reality, the two groups no longer ate lunch in the same physical spaces and morale suffered.

Under what this NP saw as an inappropriate governance regime within their PCN, NPs became mere “helpers to physicians” expected to “fill in where doctors have left holes in care,” (Participant I).

Another NP described how their role as a PCN employee had been to fill in for physicians when physicians were unavailable or during times when the physicians preferred not to work. They described how:

*Physicians [in the PCN where I worked] would not let me practice. [They] refused to allow me to practice … I didn’t get it. But then, something [would need] to be assessed right away and [they would say], “Oh, you could go do that!” After hours, Friday nights and weekends. [Then it was] no problem, but during the week I could not have clinic space (Participant XIV).*

From the perspective of a participant working for both a PCN and the provincial medical association, this employer-employee relationship along with its governance implications, was appropriate.

*[I] f we don’t have doctors that will work until 8:00 PM and NPs are willing to fill in to meet those primary care needs … [That is] TOTALLY [acceptable]. Absolutely use [the NPs] in that capacity. But for the day to day, like the eight-to-five work of the physician when we have so many physicians, it wouldn’t make sense to me that you use the NP in the same way (Participant XIII).*

A PCN administrator participant noted how deploying NPs after hours and to fill in when and where doctors were unavailable or uninterested in working was ultimately at odds with the NPSPs goal for comprehensive primary care:

*[If an NP is] just providing access in terms of evenings and weekends, you can’t necessarily be there to provide that comprehensive care (Participant IX).*

In this way the hierarchical rather than collegial governance enabled by the NPSP’s choice of the PCNs was seen, by some participants, as working against the policy’s central goals.

## Discussion

Alberta’s NPSP faces a number of critical challenges that impede its ability to achieve its stated objective of integrating NPs into the province’s primary care system. These challenges include governance issues that distribute authority and funding options unequally; financial disincentives for NPs, physicians, and PCNs; and a small number of highly delimited job opportunities. Each of these represents an opportunity to adjust the policy to be better calibrated to accomplish its main goal of NP primary care integration.

From the majority of participants’ perspectives, perhaps the most problematic aspect of the NPSP’s use of the PCNs as mechanisms of integration was that this gave physicians the final say on job availability, remuneration, and termination, as well as how key ideas like ‘care team’ are operationalized. In this sense, our interviews highlighted governance impediments to NP integration similar to those identified elsewhere [54], with other policy and legislative arrangements described as major barriers to effective integration [55]. Whether NPs are to be employees or independent providers, for primary care integration to succeed, governance arrangements that see them collaborating with - rather than subordinate to physicians - are likely a pre-requisite. Here we are drawing on the observations of others who have noted the ways in which funding and care delivery models that support medical dominance tend to impede collaboration [56]. Our data confirm that NPs are not encouraged to integrate when physicians are granted the authority to resolve territorial conflicts over scope of practice in their own favour, or to define whether a team will be collegial or hierarchical. As D’Amour et al. [57] have noted, successful collaboration in healthcare teams is the result of careful work at interpersonal, system, and governance levels, not imbalanced relationships. In this sense, the NPSP in its present form, embedded as it is in the billing and governance structures of the province, is not able to reach its full potential as a means to increase access to quality primary care through NP participation.

Beyond governance as an issue of professional autonomy, the NPSP fails to address longstanding financial disincentives that affect NPs themselves, physicians, and the PCNs. It is imperative to consider physician compensation structures in place where NPs are attempting to integrate. Family physicians in Alberta are able to directly access public funding by billing the government FFS [58], or if they choose through an annualized, sessional or blended capitation agreements with the government [41]. In contrast, the NPSP affords none of these options to NPs and instead requires them to be employees of “physician-controlled” PCNs. As such, NPs find themselves both unable to open practices of their own and find it challenging to generate enough revenue to cover the overhead they incur as employees. For their part, physicians operating on a FFS basis – which is to say the majority of primary care practices in the province – find that NPs, along with other members of the care team working alongside them, are unable to bill for services that physicians would normally provide. The inability to bill the government for services rendered by non-physician care team members has been consistently identified as an impediment to integrating non-physicians into FFS practices [26, 59]. Indeed, it has been identified as restricting NP integration specifically [54, 56, 60–63].

If these are the disincentives for individual NPs and physicians, at the PCN level the NPSP proposes that PCNs use the per capita revenue generated by the patients on an NP’s panel to cover, or partially cover, that NP’s salary. The challenge here is that this per capita funding is at the center of the PCNs’ financial model. Redirecting per capita payments – the PCNs’ only source of revenue – towards NP salaries is a redirection away from other care initiatives and practice support work at the heart of these organizations’ mandate. In this sense, deploying a physician who can bill the government FFS, and not an NP who draws down the local budget, is a more sensible option as a physician frees up more PCN money. The NPSP does not adequately address the fact PCNs are financially incentivized to utilize physicians over NPs – a critical point since the decision to utilize NPs, as the policy is presently written, remains a choice for the PCN to make. If the goal is for long-term NP utilization, rather than a “fill in” for the short-term, these financial disincentives need to be addressed. The sense among participants was that options beyond PCNs, and options beyond panel driven general practice were central to achieving greater NP integration in the province’s primary care system.

Participants made it clear that part of the shortcoming of the NPSP was in its failure to conceptualize the operational and practical role of NPs as they integrated into primary care. The Program does not provide clarity regarding the roles and positioning of NPs in the primary care system: is the objective to add NP jobs, where the NP acts as a supplement to physicians in certain geographical areas, with certain patient population, or after hours? Or is the role of the NP to partner with a physician and together manage patients? Perhaps the goal was to enable NPs to operate as independent practitioners. These goals do not have to be mutually exclusive, but each require different operational and funding barriers to be addressed for the stated goal to be met. Any policy that impacts the role of NPs should offer clear definitions of goals and roles of the program, particularly from the perspective of the finer operational details. In addition, the concerns of existing stakeholders need to be anticipated and addressed in a comprehensive manner – meaning they must be developed with broad clinician and stakeholder consultation. In other words, NPs should, as one participant put it, “have a seat at the table” in providing input to how their role is utilized.

## Conclusion

Three major factors are impeding Alberta’s NPSP from realizing its own objective of increasing NP integration into primary care: 1) financial viability issues in which NPs, physicians, and PCNs are all adversely affected; 2) policy issues in which PCNs with competing priorities act as NP employers, and NPs are expected to panel patients in competition with PCN physicians; and 3) governance issues in which NPs are not afforded sufficient authority over their role or how the key concept of ‘care team’ is defined and operationalized. In its current iteration, the NPSP does not appear to be a long-term solution for increasing NP integration into the province’s primary care environment. Increased NP integration in primary care likely requires increased funding flexibility that will allow NPs to access funding directly from the government, outside PCNs, with funding options to fit their individual practice setting. In addition, future NP policy development should: 1) ensure a clear goal for the NP role is established through clinician and stakeholder consultation including NPs themselves; and 2) ensure funding, policy, and governance structures are aligned with this envisioned goal for successful NP integration into various primary care practice settings.

## Data Availability

Recordings and transcripts of interviews are saved but not made public to protect the identity of the participants.

## Abbreviations

AMA: Alberta Medical Association
CARNA: College and Association of Registered Nurses of Alberta
FFS: Fee-For-Service
NP: Nurse Practitioner
NPAA: Nurse Practitioner Association of Alberta
NPSP: Nurse Practitioner Support Program
PCN: Primary Care Network
PHC: Primary Health Care
RN: Registered Nurse

## Footnotes

1 https://www.alberta.ca/assets/documents/health-pcn-nurse-practitioner-support-program-guide.pdf

2 Team-based care involves the collaboration of a multidisciplinary team of healthcare professionals such as physicians, nurses, nurse practitioners (NPs), dietitians, pharmacists, social workers, and mental health professionals [34]
